# Accelerated forgetting? An evaluation on the use of long-term forgetting rates in patients with memory problems

**DOI:** 10.3389/fpsyg.2015.00752

**Published:** 2015-06-09

**Authors:** Sofie Geurts, Sieberen P. van der Werf, Roy P. C. Kessels

**Affiliations:** ^1^Department of Medical Psychology, Canisius Wilhelmina HospitalNijmegen, Netherlands; ^2^Donders Institute for Brain, Cognition and Behavior, Radboud UniversityNijmegen, Netherlands; ^3^Department of Psychology, Brain and Cognition, University of AmsterdamAmsterdam, Netherlands; ^4^Department of Psychiatry and Medical Psychology, Onze Lieve Vrouwe GasthuisAmsterdam, Netherlands; ^5^Department of Medical Psychology, Radboud University Medical CenterNijmegen, Netherlands; ^6^Centre of Excellence for Korsakoff and Alchohol-Related Cognitive Disorders, Vincent van Gogh Institute for PsychiatryVenray, Netherlands

**Keywords:** long-term memory, episodic memory, amnesia, neuropsychological tests, memory consolidation

## Abstract

The main focus of this review was to evaluate whether long-term forgetting rates (delayed tests, days, to weeks, after initial learning) are more sensitive measures than standard delayed recall measures to detect memory problems in various patient groups. It has been suggested that accelerated forgetting might be characteristic for epilepsy patients, but little research has been performed in other populations. Here, we identified eleven studies in a wide range of brain injured patient groups, whose long-term forgetting patterns were compared to those of healthy controls. Signs of accelerated forgetting were found in three studies. The results of eight studies showed normal forgetting over time for the patient groups. However, most of the studies used only a recognition procedure, after optimizing initial learning. Based on these results, we recommend the use of a combined recall and recognition procedure to examine accelerated forgetting and we discuss the relevance of standard and optimized learning procedures in clinical practice.

## Introduction

Forgetfulness is a frequent complaint in patients with brain disease or brain injury Patterns of memory decline of patients may depend on the etiology. For instance, memory problems in patients with Alzheimer's dementia (AD) or Mild Cognitive Impairment (MCI) are characterized by the inability to store new memories (Pike and Savage, 2008). After stroke, memory deficits are also common, but their presentation is heterogeneous. Depending on the location and number of cerebral infarcts stroke may result in encoding and/or consolidation deficits of different domains, including verbal or visual episodic memory impairment (Lim and Alexander, 2009; Saczynski et al., 2009). After traumatic brain injury (TBI) a large range of memory aspects can be affected. Memory impairments in TBI patients particularly affect effortful encoding and retrieval processes. In contrast to severe amnesia patients, memory performances after TBI can be in the normal range (Vakil, 2007). Since memory problems occur frequently, the investigation of memory is an important part of neuropsychological assessment. Most clinically used memory tests exist of a learning procedure to facilitate encoding and storage of the target-information, followed by a delayed recall and/or recognition test. The time interval between learning and delayed testing is usually between 20 and 30 min (Lezak et al., 2014). However, it is not uncommon that patients who report memory complaints perform unimpaired on these kinds of memory tests. For instances, numerous studies have shown a lack of correlation between subjective memory complaints (SMC) and objective memory test performances (Vestberg et al., 2007; Aben et al., 2011). Consequently, it has been suggested that in these cases, the use of longer-term forgetting rates may be more sensitive to detect memory problems (Butler and Zeman, 2008).

Studies in epilepsy patients have shown that accelerated forgetting occurs after a prolonged period of time in patients who display unimpaired performances on standard memory tests. Using additional intervals varying between 24 h and 6 weeks, single-case and group studies reported evidence of accelerated long-term forgetting (Fitzgerald et al., 2013). Recently Elliot et al. (2014) reviewed the use of long-term delays in memory testing of epilepsy patients. The review discusses methodological issues and highlights the relevance of assessing accelerated forgetting. They presented seven recommendations to improve the assessment of long-term forgetting (Box 1). They conclude that accelerated forgetting is characteristic for temporal-lobe epilepsy patients. Their findings also suggest that rates of forgetting over a prolonged period of time could be an alternative and perhaps more sensitive measure for perceived memory problems than short delays.

Box 1 **Methodological recommendations for assessing long-term forgetting (Elliot et al., 2014).**

1 Patients and controls should be matched on age and general cognitive functioning/educational background
2 Verbal and non-verbal tests should be used
3 Ideally, recall and cued recall/recognition procedures should be used
4 Ceiling and floor effects should be avoided
5 The potential for rehearsal and repeated recall should be avoided
6 To prevent that immediate recall can rely on short term memory it must be ensured that information is stored in long-term memory. A filled delay of at least10s before immediate testing is recommended, to ensure that information is retrieved from long-term memory on both time points
7 Efforts should be made to equate initial learning

Their findings are in agreement with the active and long lasting process of memory consolidation, that is, the theoretical process that ensures a stronger representation of memories over time. Findings on retrograde amnesia suggested the existence of a gradual process of reorganization of memory, also known as the consolidation process (McGaugh, 2000). The exact time-frame of memory consolidation is, however, under debate, yet it is assumed that the formation of stable memory presentations may continue for months or even years (Gold, 2006). Squire (1986, 2009) emphasized in several reviews the temporary role of the hippocampus in the formation and retention of memories and suggested two consolidation processes: a fast consolidation process mediated via medial temporal-lobe structures, and a slow consolidation process mediated via a repeatedly activated hippocampal-neocortical connection. The key concept of what is known as the “standard consolidation theory” is that memories become independent of the hippocampal region (Squire and Alvarez, 1995). The alternative “multiple trace theory” provides a different view and makes a distinction between episodic and semantic memories. According to this theory the hippocampal area and neocortex continues to be involved in the storage and retrieval of episodic memories. In contrast semantic memory becomes represented outside the hippocampal region in neocortical structures (Nadel and Moscovitch, 1997).

In summary, both clinical and neurobiological findings suggest that assessing the outcome of memory consolidation over a prolonged period could be useful in clinical practice. However, little is known about the existence of accelerated forgetting in various patient groups. Since in clinical practice memory complaints may not always be detected in standard memory tests, the aim of this review is to summarize the findings of long-term forgetting rates in patient groups other than epilepsy patient and to discuss the possible existence of accelerated forgetting within these groups. In line with Elliot et al. (2014) our second aim is to provide recommendations for the use of long-term forgetting rates in non-epilepsy groups.

## Method

A literature search was conducted in Medline and Pubmed to provide an overview of studies addressing forgetfulness while applying memory tests with extended delayed recall conditions and focusing on patient groups other than epilepsy patients. We thereby focused on studies that used well-known tests or procedures and compared patient groups with healthy controls. The last search took place on January 14, 2015. The following words were used: *long-term forgetting, accelerated long-term forgetting, forgetting rates, abnormal forgetting, long-term amnesia*, and *long-term memory consolidation*. The electronic search was supplemented by searching the reference lists and by contact with other investigators. The selection was discussed among the authors until consensus was reached.

The following inclusion criteria were applied: (1) had to be published in the English language; (2) pertained to the assessment of patients of 18 years or older; (3) presented a controlled design, using healthy controls as a comparison group; (4) used episodic memory tasks; (5) the presence of a standard delayed test interval (generally 20–30 min) and (6) an additional long-term delayed test interval with a minimum of 24 h. Papers that focused on epilepsy patients were excluded, as findings of this patient group were already reviewed by Elliot et al. (2014). Also, studies focusing on normal aging (e.g., comparing healthy older adults with healthy younger adults) were excluded.

## Results

The initial search yielded in total 138 studies (a flowchart is provided in Appendix 1). Of these, 129 studies were excluded after selection based on their title and abstract. Main reasons for exclusion were: epilepsy (*N* = 29), animal studies (*N* = 25), other kinds of memory test procedures, e.g., implicit memory tests, priming-experiments, or studies assessing familiarity vs. recollection (*N* = 19), studies in healthy participants only, e.g., aging studies (*N* = 18). Of the remaining number, five more studies were excluded after close reading, because the additional delayed test interval was shorter than 24 h or specific experimental testing procedures (such as differences in memory of emotionally highly or neutral words) were used. This led to a remaining number of three studies. Eight studies were included through manually screening of the reference lists and by contact with other investigators. One study by Squire (1981) appeared to fulfill all criteria, except that this study used control groups of non-healthy participants. In total, 11 studies met all inclusion criteria and relevant characteristics of the selected studies have been presented in Table 1. The studies are subdivided and discussed according to patient group. In each paragraph we describe the methods that were used and summarize the findings and conclusions of the authors.

**Table 1:** **Characteristics of the studies**.

**Authors**	**Patients (N)**	**Controls (N)**	**Learning procedure and initial level comparable?**	**Interval**	**Test**	**Method**	**Result ALF present Yes/No**	No. of criteria met (Elliot et al. 2014)^*^
Huppert and Piercy, 1978	KS (7)	7	Optimized Yes	10 min; 1 day; 7 days	120 pictures H&P method	Recognition	No	3
McKee and Squire, 1992	MTL damage (5) DC damage (6)	10	Optimized Yes	10 min; 2 h; 32 h	120 Pictures H&P method	Recognition	No	4
Lewis and Kopelman, 1998	ECT (13) Delirium (8) Depr. non-ECT (16) Schizophrenia (4)	5	Optimized Yes	10 min; 2 h; 24 h	120 Pictures H&P method	Recognition	No	3
Levin et al., 1988	TBI in PTA (13) TBI out PTA (18)	18	Optimized Yes	10 min; 2 h; 32 h	66 Pictures H&P method	Recognition	No	4
Spikman et al., 1995	TBI out PTA (22) MCI (14)	15	Standard Yes	1–2 h; 3 weeks; 6 weeks; 27 weeks	100 pictures Eq. H&P method	Recognition	No	3
Kopelman, 1985	AD (16) KS (16)	16	Optimized Yes	10 min; 24 h; 1 week	120 Pictures H&P method	Recognition	No	4
Carlesimo et al., 1995	AD (13) VaD (8) Amn (9)	12	Optimized Yes	90 s; 10 min; 1 h; 24 h	100 pictures Eq. H&P method	Recognition	AD: Yes Amn: Yes MID: No	4
Manes et al., 2008	MCI (5) SMC (10)	9	Standard No: MCI < SMC and controls Yes: SMC = controls	30 min; 6 weeks	2 Stories RCFT	Recall	MCI: No SMC: Yes	4
Walsh et al., 2014	MCI (15)	15	Optimized	30 min; 1 week	1 Story	Recall	Yes	4
DeLuca et al., 1998	MS (40)	20	Optimized Yes	30 min; 90 min; 1 week	10 Word list	Recall and Recognition	No	4
Gaudino et al., 2001	MS (64)	20	Optimized Yes	30 min; 90 min; 1 week	10 Word list	Recall and Recognition	No	4

KS, Korsakoff's syndrome; MTL, medial temporal lobe; DC, diencephalon; ECT, electroconvulsive therapy; TBI, traumatic brain injury; PTA, post traumatic amnesia; SCI, subjective memory impairment; AD, Alzheimer disease; VaD, vascular dementia; Amn, amnesics; MCI, mild cognitive impairment; SMC, subjective memory complaints; MS, multiple sclerosis; H&P, Huppert and Piercy; RCFT, Rey complex figure test; ALF, accelerated forgetting;

* *, with a maximum score of 6 after exclusion of criteria 4*.

### Long-term forgetting rates in medial temporal lobe and diencephalon amnesic patients

The most widely used method to assess long-term forgetting rates was introduced by Huppert and Piercy in the research into the etiology of amnesia (Huppert and Piercy, 1978). The method they described tests recognition memory of 120 slides and tries to minimize initial learning impairment by enlarge the presentation times of the slides within the patient groups. 10 min after presentation of the slides, the participants have to recognize 40 of the original targets among 40 distracters. After delayed intervals (varying from 24 h to 7 days, depending on the study) the same procedure follows with 40 new targets and 40 different distracters. Huppert and Piercy tested seven Korsakoff patients and six controls using intervals of 1 and 7 days. The rate of decline did not differ between the groups and they concluded that Korsakoff patients have a deficit in learning performances, yet forget normally over time.

In line with this, McKee and Squire (1992) applied the Huppert and Piercy method trying to distinguish medial temporal lobe (MTL) from diencephalic memory profiles. They examined five amnesic patients with MTL damage, six amnesic patients with diencephalic damage and 10 matched controls. The main forgetting rates after 2 and after 32 h did not differ significantly for the three groups. The authors concluded that differences in the localization of the structural cerebral damage were not associated with differences in long-term forgetting patterns. Recent findings are in agreement with their conclusion. The last years it is demonstrated that the diencephalon is involved in both encoding and retrieval processes and that the explicit memory system relies on a neural diencephalon-hippocampal circuit (Kessels and Kopelman, 2012; Kopelman, 2014).

Lewis and Kopelman (1998) suggested an alternative cause for the various long-term forgetting patterns. They hypothesized that metabolic disruption may lead to long-term forgetting instead of structural lesions. They administered the Huppert and Piercy method with intervals of 10 min, 2 and 24 h, to 13 electroconvulsive therapy patients (ECT) and eight delirium patients (both presumed for metabolic disruption), 16 non-ECT depressed patients (to control for depression), four schizophrenic patients (assuming MTL dysfunction) and 5 healthy controls. Over the period between 2 and 24 h no significant difference in rate of forgetting was found between the groups.

### Summary

All three studies applied a recognition memory test, in which initial learning levels of the patients and controls were equated. Forgetting rates were tested after 24 h, 32 h, and 7 days. None of the studies found signs of accelerated forgetting. The patient groups forget in a similar rate after initial learning was optimized compared to controls.

### Traumatic brain injury patients: during and after a period of PTA

Levin et al. (1988) followed the hypothesis of two distinct forms of amnesia that were related to different brain structures. Assuming a relation between traumatic brain injury (TBI) patients and temporal-lobe damage, they examined long-term forgetting patterns of 13 TBI patients during PTA, 18 TBI patients after recovery of PTA and 18 controls. Again the Huppert and Piercy method was used, this time using 66 slides. The results showed that in subsequent time periods (2 and 32 h), the three groups had comparable forgetting rates. Since patients during PTA had more severe injury characteristics compared to the patients after recovery of PTA, the authors matched the injury severity of both patients groups. The matching did not alter the previous findings and therefore they concluded that TBI might impair the acquisition of new information but not necessarily the consolidation.

Spikman et al. (1995) also examined long-term forgetting rates of TBI patients. They investigated whether the ability of recognizing information was unimpaired in groups with clear evidence of memory impairment. In addition to 22 chronic TBI patients, they included 14 healthy elderly with memory deficits without dementia (Nowadays mild cognitive impairment “MCI”) and 15 healthy controls. In addition to three regular memory tasks, they designed the 100 Pictures Test which is similar to the Huppert and Piercy method. After presenting 100 slides, an immediate forced choice recognition test was applied with 20 targets and 20 distracters. Four more recognition sessions followed with 20 other targets and distracters. The intervals were: 1–2 h, 3, 6, and 27 weeks. In contrast to the Huppert and Piercy method (Spikman et al., 1995) did not use an optimized learning procedure. However, the results showed comparable performances on the immediate test. Despite flawed results of the TBI patients and elderly (MCI) on the regular memory tests, all groups showed comparable forgetting rates up to 27 weeks on the recognition test.

#### Summary

The previous two studies in traumatic brain injury patients showed that accelerated forgetting did not occur when recognizing visual information. Even the presence of PTA did not lead to faster forgetting over time, provided that initial learning was optimized.

### Accelerated forgetting in dementia and MCI

Following previous research on accelerated long-term forgetting in amnesia Kopelman (1985) compared the long-term forgetting rates of 16 AD patients, 16 Korsakoff patients, and 16 healthy controls. Given the severe atrophy of the medial temporal lobe in Alzheimer patients, it was expected that this group showed enlarged forgetting rates compared to Korsakoff patients and controls. The Huppert and Piercy paradigm was used, with intervals of 24 h and 7 days. Kopelman (1985) found that once initial learning had been equated, the AD and Korsakoff groups displayed similar long-term forgetting rates as the control group. The author concluded that differences in delayed recognition between healthy controls, AD patients and Korsakoff patients were primarily due to impaired acquisition.

Carlesimo et al. (1995) aimed to replicate findings on long-term forgetting in Alzheimer patients and tested in addition long-term forgetting rates in vascular dementia and other kind of amnesic patients. They tested 13 AD patients, 8 vascular dementia (VaD) patients, 9 amnesic patients (amnesia was developed as a consequence of traumatic brain injury or stroke), 11 controls for the dementia groups, and 12 controls for the amnesic group. A modified version of the Huppert and Piercy method was applied, in which after 90 s a first recognition test followed. Participants who did not recognize 80% correct were again exposed to the stimuli set and were excluded when they did not reach criterion the second time. Subsequent delayed intervals of 10 min, 1 and 24 h were used. Within their conclusion the authors argued that between 1 and 24 h the AD patient and amnesics forget in a faster way compared to the controls, but that the VaD patients forget in a normal rate. However, they provided no statistical data concerning these conclusions, so some cautions should be made when interpreting these findings.

More recently Manes et al. (2008) examined whether accelerated forgetting is an early sign of MCI, which is often considered the pre-dementia stage of Alzheimer's disease. They compared long-term forgetting rates of MCI patients with those of patients with SMC and age-matched controls. All participants were presented with two short stories and were required to copy the Rey complex figure. The SMC patients and the healthy controls displayed similar performances on all the immediate and 30-min delayed recall measures and outperformed the MCI patients. However, after a 6-week delay, both groups of patients had mean memory scores significantly below those of the healthy control group and the two patient groups became indistinguishable.

The long term forgetting rates of 15 MCI patients and 15 healthy controls were also examined by Walsh et al. (2014), using a story recall task. In contrast to the study of Manes et al. (2008), they matched initial acquisition by using an optimized learning procedure. All participants had to recall the story after each presentation until a criterion of 90% correct was reached, with a minimum of five trials. Delayed recall was tested after 30 min and after 1 week. On both time points the MCI patients performed worse in comparison to the controls and between the 30 min and 1 week period the rate of forgetting of the MCI patients increased. The authors discussed that the use of a 30-min delayed recall task could result in an underestimation of the memory problems of MCI patients.

#### Summary

The two dementia studies above applied the Huppert and Piercy method using an optimized learning method and intervals up to 7 days. The results of the studies are conflicting, which makes it unclear whether long-term forgetting of visual recognition within dementia patients does occur. The two MCI studies examined long-term forgetting rates using a verbal recall test. Both studies found signs of accelerated forgetting after 1 week. The most recent study matched for initial learning level and even then faster forgetting for MCI patients was found.

### Patterns of long-term forgetting in multiple sclerosis

DeLuca et al. (1998) investigated whether memory impairments in multiple sclerosis (MS) were due to deficits in acquisition or retrieval. A word list learning task was administered in 40 MS patients and 20 controls. They had to learn a list of 10 semantically related words, during a maximum of 15 trials. Only words that were not recalled by the participant were repeated until on two consecutive trials all 10 words were recalled correctly. Delayed free recall and recognition were tested after 30 min, 90 min, and 1 week. In the recognition task participants had to recognize the target words among a list of 40 words. The MS patient group needed more learning trials compared to the controls, but after 1 week the rate of forgetting did not differ between the groups. The authors concluded that the memory impairment of MS patients was due to acquisition problems and not to faster forgetting.

Gaudino et al. (2001) examined learning performances and long-term forgetting in patients with multiple sclerosis (MS). They compared 64 MS patients with 20 healthy controls and administered a word list learning test, as part of a comprehensive neuropsychological assessment, with an optimizing learning procedure using the same method as the study described above. The recall and recognition scores declined across the three intervals (30 min, 90 min, 1 week), but the patterns of decline for MS and the control group were comparable.

#### Summary

Both studies in MS patients facilitated the learning performances and tested long-term forgetting via the same method using a verbal recall and recognition test. The results indicate that patients with MS do not show accelerated forgetting on longer term.

## Discussion

Measuring long-term forgetting has been suggested to be potentially useful in clinical practice. While most studies using long-term forgetting rates have been applied in epilepsy patients, this review aimed to provide an overview of studies investigating long-term forgetting in other patient groups. Our goal was to establish whether these rates would be useful for examining patients who report memory complaints, yet perform normally on regular memory tests. Our search revealed that only a few studies used extended delayed intervals in different patient groups. Strikingly, only three studies demonstrated convincing evidence for accelerated forgetting for (a subgroup) of their study population. Based on these findings there is no convincing evidence that accelerated forgetting is a characteristic in non-epilepsy patient groups suspected of having memory deficits, but methodological issues and the small number of studies make strong conclusions difficult.

### Patient groups

Faster forgetting has been demonstrated in a group with AD, a group of MCI patients and a group of older adults with SMC who did not fulfill the criteria for MCI. In contrast, another study with Alzheimer patients showed normal forgetting over time. The results regarding this patient group are therefore inconclusive. For all other patient groups the studies showed normal forgetting rates over time in comparison to healthy controls. This involved patient groups with amnesia (of MTL or diencephalic nature), TBI, MS, and neuropsychiatric disorders (ECT-treated and non-ECT treated depression, delirium, Korsakoff's syndrome, schizophrenia). Since only a limited number of studies were found, the results should be interpreted with caution, but the findings provided no convincing evidence that accelerated forgetting is a characteristic in these patient groups. In epilepsy patients more evidence for faster long-term forgetting has been demonstrated (Elliot et al., 2014). Possibly, epilepsy-related variables may have contributed to accelerated forgetting in these patients, but studies addressing the association between accelerated forgetting and epilepsy variables, including interferences of seizures and anti-epileptic drugs, showed conflicting results (Fitzgerald et al., 2013). Other epilepsy studies suggested a critical role for the temporal lobe, whilst other studies could not relate accelerated forgetting to hippocampal abnormalities (Muhlert et al., 2011; Lah et al., 2014). The latter is in agreement with findings of this review. That is, several studies used clinical groups with temporal lobe damage (amnesic patients, Alzheimer demented patients) but showed similar forgetting rates compared to controls. Although accelerated forgetting seems to be more common in epilepsy patients than in other patient groups, it remains an open question what the possible contributors are.

### Test material and procedures

Seven out of eleven studies used the method described by Huppert and Piercy or an adaptation of this method. This means that a large majority of the studies assessed long-term forgetting via a visual recognition test after optimizing initial learning. Only four studies used a free-recall procedure. Our review shows that the ability to recognize information over prolonged time is highly robust over time. In general, recognition tests are seen as less effortful and susceptible for ceiling effects. The low level of attention and mnemonic processing that is needed to recognize previously presented information could perhaps contribute to this insensitivity to change over time. In contrast, active retrieving information from memory, as in free-recall procedures, demands more cognitive effort, which makes it more susceptible to impairments. Complaints of everyday forgetting usually refer to the inability to recall certain information. As a result, using only a recognition procedure does not resemble the mnemonic demands of everyday life.

As discussed earlier, Elliot et al. (2014) described methodological recommendations for assessing long-term forgetting (Box 1). From the included studies in our review, it is unclear whether they accounted for the recommendation to avoid possible floor or ceiling effects, but the other six recommendations could be evaluated. In general, criterion 6—apply a filled delay of at least 10 s after learning before immediate recall—is not fulfilled in the studies that used existing clinical memory tests. However, in those cases long-term forgetting rates were determined on a 30-min delay plus an additional long-term delay, which also prevents the use of short-term memory processes. Overall, the methods of the 11 included studies fulfilled only three or four of the recommendations. Hence, we conclude that the methods of most of the included studies are limited.

Although we endorse the recommendations of Elliot et al. (2014), we suggest two additional recommendations based on our findings. As explained above, the use of only a recognition test increases the risk of ceiling effects. We propose to use their third recommendation more strictly and suggest that the combination of a recall and recognition procedure is a prerequisite for detecting long-term memory problems. Results of a recent epilepsy study are in line with this. Hoefeijzers et al. (2013) demonstrated faster forgetting for verbal recall in epilepsy patients compared to controls, but demonstrated comparable long-term forgetting rates for picture recognition. In this study, long-term memory problems should not have been detected when only a recognition procedure was applied. Furthermore, our review shows that when initial learning is successful, acquired memories are preserved over the longer term. For many years there has been a debate about how to compare forgetting rates when levels of original learning are not similar between groups (Loftus, 1985; Wheeler et al., 2003). Several techniques have been developed to adjust for initial learning difficulties, but no consensus exists on whether and how this should be done (Wheeler, 2000). It can be questioned whether results of memory tests after optimized initial learning reflect actual learning and memory in daily situations. That is, in daily life it is not common that information is repeatedly presented. Although we agree that the consolidation of memories can only be “purely” evaluated after controlling for learning difficulties, we argue that in addition to optimized learning procedures it is important to assess standard learning as well as to resemble the demands of daily life at most. Long-term forgetting rates after standard learning methods presumably reflect the memory complaint better, while long-term forgetting rates after optimized learning levels provide relevant information on the nature of the memory problem and for treatment possibilities.

### General conclusions and future directions

In this review we evaluated whether patients with memory deficits due to a wide variety of etiology show evidence of accelerated forgetting using long-term (i.e., days–weeks) delayed testing intervals. Eight out of the eleven included studies found normal long-term forgetting patterns in patients compared to healthy controls, provided that initial learning was equated. In agreement with evidence for accelerated forgetting in epilepsy patients (Elliot et al., 2014), three studies demonstrated faster long-term forgetting for patients with (presumed) MCI and AD in comparison to healthy controls. So far, it remains an open question what the possible contributors are. Our review highlights the importance of the use of a combined recall and recognition procedure to detect accelerated forgetting and we have advocated the relevance of both an optimized and a standard learning procedure in clinical practice.

A few limitations of this review should be mentioned. First of all we included only papers written in English, therefore published studies written in other languages are missing. In addition, we included studies with well-known memory tests. Studies with experimental tests were excluded, thus results from these studies are lacking. Also, we focused on studies comparing patients to healthy controls and we excluded studies focusing on long-term forgetting in normal aging, as these typically compare healthy older adults with young adults in a cross-sectional manner. Nowadays, early detection of MCI is considered relevant and the need for sensitive measures to detect mild memory problems is growing. Although we aimed to review patient-based findings and normal aging was beyond our scope, it is of interest for future studies to consider the results of long-term forgetting in normal aging within this context, preferably focusing on studies using longitudinal designs.

Based on our review a number of recommendations can be postulated. Since, conclusions so far are limited to visual recognition procedures, future studies should use more sensitive and diverse test procedures. Combining recall and recognition procedures, as well as visual and verbal memory tests, may be more sensitive to demonstrate accelerated forgetting in patients with memory complaints. Second, for future research we recommend to address the distinction between contextually bound and context-free memory tasks. Research in the realm of memory consolidation and the multiple trace theory, suggests that contextually bound information (episodic information), is mediated primarily by the MTL and thereby is impaired when the hippocampus is damaged regardless of the age of the memory. Context-free information may be independent of hippocampal structures as the consolidation process continues and across time is less affected by hippocampal dysfunctioning (Winocur et al., 2010). Consistent with this view Tramoni et al. (2011) reported steeper forgetting rates for contextually bound information in a group of epilepsy patients after a delay of 3 weeks, in contrast to the memory performance of context-free information that was preserved. For future studies it is interesting to consider whether the degree of context explains the occurrence of accelerated forgetting.

### Conflict of interest statement

The authors declare that the research was conducted in the absence of any commercial or financial relationships that could be construed as a potential conflict of interest.

## Figures and Tables

**Figure:**
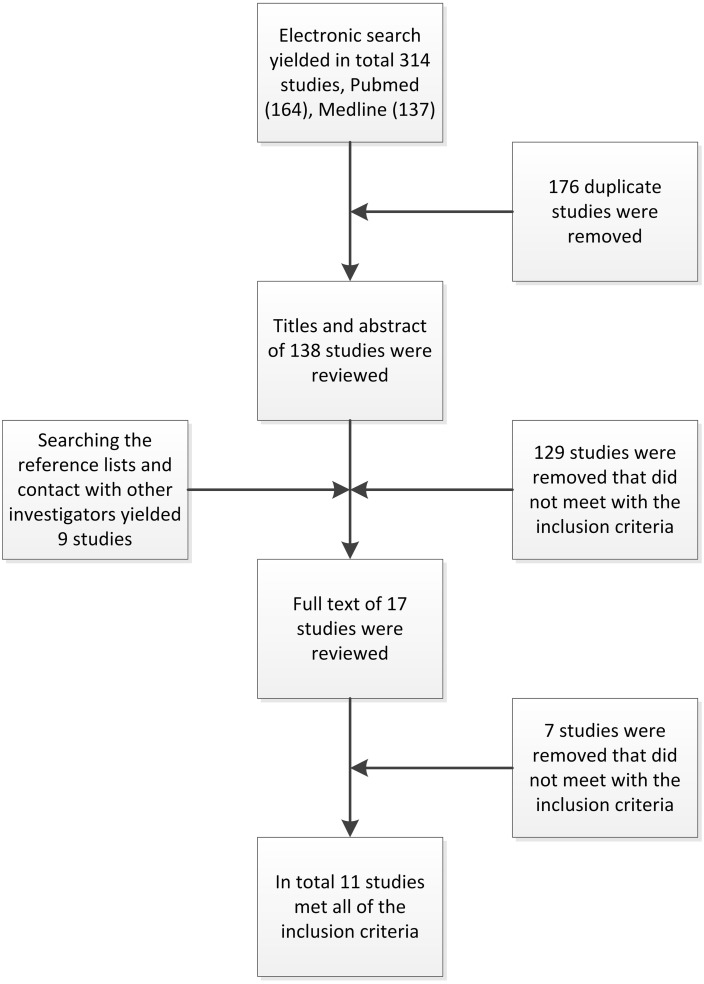
Flowchart of the literature search
